# The Opening of the Princess Alice Home, Middlesex Hospital

**Published:** 1920-07-17

**Authors:** 


### THE OPENING OF THE PRINCESS ALICE HOME, MIDDLESEX HOSPITAL.

THE Clerk of the Weather decided to be fairly kind to the Middlesex Hospital on the occasion of the opening of the Princess Alice Nurses' Home on July 8. The sun burst out about three o'clock after one of many showers, and lighted up the pretty exterior of the building as the guests began to arrive. Miss Montgomery, the matron, had arranged that everyone should be able to go round the Home, and had posted nurses along the "line of route" to explain arrangements and answer questions. The Middlesex nurses are inspired by the same spirit of hospitality as their matron, and sisters, staff nurses, and probationers alike made charming assistant hostesses.

The Home is intended for the accommodation of the night staff only. A separate building for night nurses is, of course, an immense advantage to the staff of a hospital. The rooms and passages are all papered in soft grey, and the walls above the deep picture-rail are coloured white; the floors are covered with a particularly pretty shade of green linoleum. A bright rug in each room gives the necessary note of colour. The furniture is dark fumed oak, and the quilts dark blue and white. The picture-rails are a very important point. Pictures give the individual touch to a room which makes it homelike, and it seems hard not to allow them, yet nails knocked in at irregular intervals on the walls are unsightly. Another delightful arrangement is that of a kettle and ring for making tea, one on each floor of the Home, near the bathrooms. The Home is warmed by radiators, but there are fireplaces in some of the larger rooms. The sitting-rooms on the ground floor have the same pleasant restful colouring as the bedrooms, but the comfortable couches and armchairs are upholstered in pretty shades of dark mauve. Both couches and chairs look as if they were really meant to be used, and to afford real rest to tired nurses. Behind the Home is a good-sized piece of ground, one portion laid out as a badminton court in red shale, the rest grass. This will be a pleasant retreat, and a place in which nurses can feel free, and not overlooked by the wards as the hospital garden is.

The proceedings at the opening were pleasantly informal. Princess Alice and the Earl of Athlone were received by the Duke of Northumberland (President of the Hospital) and Miss Montgomery (Matron), and conducted to one of the sitting-rooms. Here the Princess declared the Home open, accepted a bouquet of pink carnations presented by a junior night nurse, and consented to be photographed with the guests and as many of the staff as possible. The Princess then went through the rooms and inspected all the arrangements.

After the ceremony the guests proceeded to the hospital, where a garden-party was held. Tea was served in the board room, where the Wayfarers' Amateur Orchestra played most delightfully. The nurses and their friends had tea in the large refectory looking out on the hospital garden. Very interesting demonstrations were given in the theatre and research departments later in the afternoon.

